# The emperor's new clothes: hypersensitivity of the new cardiac isoenzymes

**DOI:** 10.3402/jchimp.v3i1.20354

**Published:** 2013-04-17

**Authors:** Ma Ai Thanda Han, Vivek Cherian, R. Dobbin Chow

**Affiliations:** 1 Department of Medicine, Medstar Good Samaritan Hospital, Baltimore, MD, USA; 2 Ross University School of Medicine, New Jersey, United States

**Keywords:** right ventricular myocardial infarction, pulmonary embolism, cardiac troponins, conventional troponin assays, highly sensitive troponin assays

## Abstract

Right ventricular (RV) myocardial infarction (MI) and pulmonary embolism (PE) are commonly recognized as two of the most challenging and vexing entities in clinical practice. When either is considered in a differential diagnosis, they warrant close consideration because of the life-threatening nature of these conditions. Their signs and symptoms overlap and, on rare occasions, they both can be simultaneously present in a single patient. Cardiac troponins are considered reliable markers of myocardial injury and are critical to the diagnosis of acute coronary syndromes. However, they can also be elevated in cases of PE. We herewith present a case of a woman who initially presented with syncope and then subsequently dyspnea. She manifested elevated cardiac isoenzymes, right-sided electrocardiogram abnormalities, and RV hypokinesis on echocardiography. She was initially diagnosed with RV infarct and managed with an interventional cardiology approach. However, her symptom of dyspnea persisted and the patient was eventually diagnosed with PE. Clinicians should entertain the diagnosis of PE in patients with elevated troponin I and evidence of right-sided cardiac compromise.

Acute pulmonary embolism (PE) is one of the leading causes of cardiovascular death, with a mortality rate of 15% in high-risk groups (1). Timely initiation of appropriate treatment reduces the mortality to 2–8% (2). Thus, early and accurate diagnosis of PE is critical and should be pursued through a rigorous process of clinical evaluation, concomitant risk stratification, and clinical probability assessment. As we shall see, the clinical presentation of right ventricular (RV) infarction can mimic PE. Cardiac troponins (cTns), measured as specific markers for myocardial injury, include cardiac troponin I (cTnI) and T (cTnT). There are various immunoassays to detect these cTn, but these assays vary in their sensitivity and specificity (3, 4). Newer assays, called highly sensitive assays, are being developed but are currently not widely available. cTnI and cTnT are elevated in both RV infarction and PE, thereby resulting in one diagnosis potentially being mistaken for the other. To illustrate this conundrum, we report a case of suspected RV and inferior wall myocardial infarction (MI), resulting in a delayed diagnosis of PE in a patient with elevated troponin I.

## Case presentation

A 77-year-old woman with a prior history of hypertension and hyperlipidemia was in her usual state of health until the evening of the day of admission, when she experienced dizziness, which was immediately followed by syncope. She had walked to her bedroom after hanging some decorations in her home in preparation for the Christmas holiday. She fell to the floor of her bedroom and was subsequently found by a family member. When she awoke, she complained of dyspnea. By the time she was brought to the hospital by emergency ambulance, she was asymptomatic and back to her baseline. She was a non-smoker and did not have a family history of premature coronary artery disease. Nifedipine XR was her only medication. Hyperlipidemia was controlled by diet alone.

**Fig. 1:**
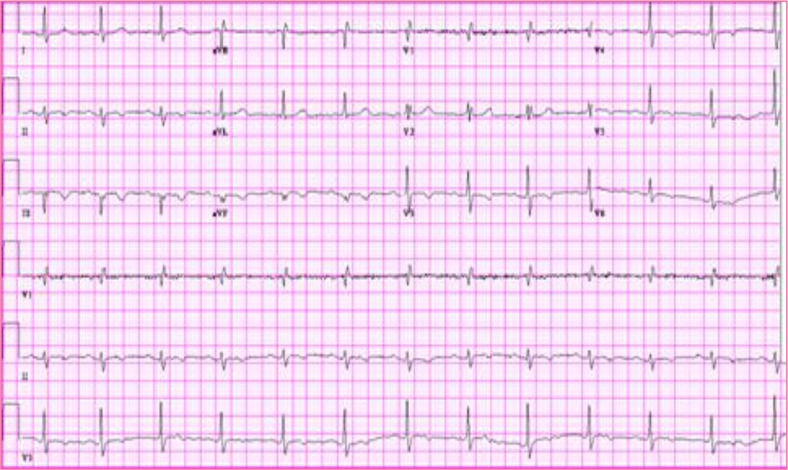
showed QS patter with T-wave inversion in III and a VF.

On initial evaluation, she was lying comfortably in no acute distress. Her BP was 126/70 mm Hg, PR 65/min, RR 18/min, oxygen saturation 100% on 2 L N/C, and temperature 36.5°C. There was no evidence of trauma. Heart sounds were regular without murmur and the apex was non-displaced. Lungs were clear to auscultation in all lung fields. The remainder of the physical examination was normal. The EKG (Fig. 1) showed a QS pattern with T-wave inversion in III and a VF. T-wave inversions were also present from V3 to V6 on right-sided EKG. Troponin I increased from 0.639 to 1.760 to 2.75 ng/ml and CK-MB 5.3 to 6.1 ng/ml over the course of the first hospital day. A transthoracic echocardiogram reported moderate hypokinesis of the right ventricle and the basal segments of the inferoposterior wall of the left ventricle; the right ventricle was dilated, with estimated RV systolic pressure of 40–45 mmHg. RV and inferior wall infarction was diagnosed and cardiac catheterization was then performed. It uncovered two 80% eccentric stenotic lesions on RAO projection of the right coronary artery, resulting in the placement of two intracoronary stents at those locations.

**Fig. 2:**
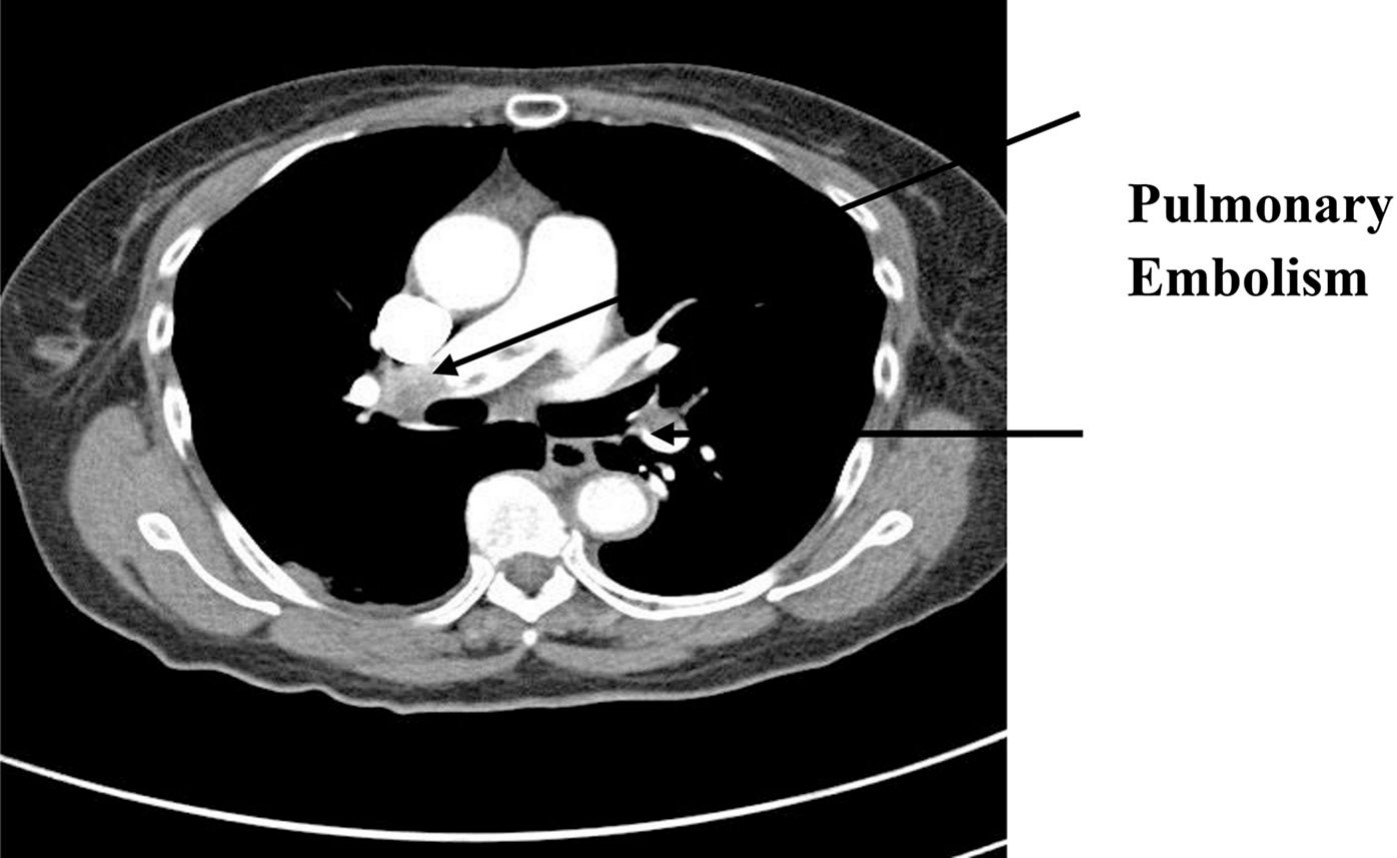
CT angiogram showing bilateral pulmonary emboli.

The patient was discharged on the second hospital day, though she complained of dyspnea with ambulation. The dyspnea, not associated with chest pain or diaphoresis, persisted at home and she returned to the hospital on the same day of discharge. Her vital signs were stable with O_2_ saturation 97% on room air. Troponin I, CK-MB and D-dimer were all elevated at 0.86, 1.33, and 4315 ng/ml, respectively. A CT angiogram (Fig. 2) showed bilateral PE with a pulmonary infarct in the superior segment of the right upper lobe. A subsequent abdominal CT revealed a thrombus in a dilated infra-renal segment of the inferior vena cava, and a bilateral lower extremity duplex ultrasound uncovered the presence of right femoral and popliteal deep vein thromboses. Anticoagulation with warfarin was started, bridging with heparin. Once the INR became therapeutic, she was discharged.

## Discussion

In a commonly referenced 1977 editorial entitled ‘Overdiagnosis and overtreatment of PE: The emperor may have no clothes’ (5), it was acknowledged that the diagnosis of PE is vexing and overdiagnosed. With the advent of the new high-sensitivity cardiac isoenzymes, which are increased in both RV infarction and PE, the diagnoses of PE and MI can be easily confused. cTn have 39% sensitivity and 93% specificity for single elevation, and 96% sensitivity and 100% specificity for serial elevation for MI (6–8). However, cTn are elevated in 0–35 and 50% of patients with non-massive, submassive, and clinically massive PE, respectively, but the sensitivity and diagnostic values are low (1, 9, 10). Neither electrocardiogram nor echocardiography easily differentiates between the two diagnoses.

The reason for elevated cTn in the setting of acute PE is still unclear. The acute increase in pulmonary artery pressure, leading to acute RV strain, has been proposed as an etiology by Meyer et al. (11). In a prospective, double-blind study of 36 patients with acute PE, they found that 62.5% of the study patients with RV dilation had an increased cTnI level, whereas 28.6% of those with positive cTnI level had a normal RV end-diastolic diameter. They concluded that positive cTnI were significantly associated with RV dilation (*p*=0.009) and more segmental defects on ventilation/perfusion lung scanning when compared to normal cTnI cases (*p*=0.0002). Other potential mechanisms for elevated cTn in PE patients include hypoxemia due to ventilation–perfusion mismatch, hypo-perfusion as a consequence of low output and reduced coronary blood flow and paradoxical embolism from systemic veins to the coronary arteries via a patent foramen ovale (12).

In autopsies of patients who have died of massive PE, transmural RV infarctions have been reported despite patent coronary arteries (13). Studies investigating the kinetics of release of cTnT in patients with PE showed that peak cTnT was lower and persisted for a shorter period of time than when compared with cTnT values in acute MI (14). This finding suggests that the mechanism of myocardial injury and the release of cTnT in patients with significant PE are significantly different from that in ACS. Several studies have reported an association between elevated troponin levels and a worse prognosis in patients with PE. Becattni et al. (15) performed a meta-analysis of 20 studies in 1,985 patients with PE. Elevated cTn levels were significantly associated with higher short-term mortality in a subgroup of hemodynamically stable patients. Another more recent meta-analysis focused on normotensive patients with acute symptomatic PE (16). In this analysis, consisting of 1,366 patients, elevated troponin levels resulted in a four-fold increase at risk of short-term death.

Conventional troponin assays are characterized by inadequate precision at the lower detection limit. As a consequence of the low prognostic sensitivity and negative predictive value of these assays, risk assessment in patients with acute chest pain (15) and in those with confirmed acute PE (16) may require serial troponin measurements. To overcome these limitations, a new-generation high-sensitive troponin assays were developed, which are capable of defining the 99th percentile of a normal (healthy) reference population with a coefficient of variation of <10% (17). Recent studies in patients with acute MI indicated an excellent diagnostic performance of these assays and the potential to improve early risk stratification (18, 19). In a study by Lankeit et al. the use of a highly sensitive TnT assay may improve risk stratification of patients with acute PE (20). Patients with high values of highly sensitive TnT have an adverse 30 days outcome for the combined endpoints of death, need for catecholamines, endotracheal intubation, or cardiopulmonary resuscitation (*p*≤ 0.027). Use of this assay may also help differentiate low-risk patients with PE who may be potential candidates for home treatment. Conversely, patients who have an elevated risk of adverse early outcomes, especially when evidence of right heart strain is present on echocardiography, may warrant closer long-term follow-up because of an increased risk of late mortality (20). A multicenter, multinational cohort study conducted by Lankeit et al. (21) suggests that the combination of highly sensitive Troponin T assay and simplified PE Severity Index (sPESI) may yield additive information for identifying low risk PE patients. They found that patients with sPESI of 0 and highly sensitive TnT of less than 14 pg/ml have a 42% reduction in their risk of death during a 6 month follow-up period in comparison with those with sPESI of ≥ 1 and/ or highly sensitive TnT of ≥ 14 pg/ml. Potentially, selected low risk patients with a PE might be safely treated in an outpatient setting. Finally, in a recent study by Walter et al. patients with acute PE and serum highly sensitive TnI values >0.1 ng/ml showed significantly higher D-dimer concentrations (*p*=0.0467) and a 5-fold increased risk of an adverse clinical outcome (odds ratio, 4.9; 95% confidence interval, 1.28–18.66; *p*=0.0235) compared with patients with acute PE and highly sensitive TnI values <0.1 ng/ml (22). Though more studies regarding the predictive value of highly sensitive troponin assay for the outcome of patients with acute PE are needed, these initial results suggest that the highly sensitive troponin assay may have both important diagnostic and prognostic utility.

## Conclusion

Clinicians should have a high level of suspicion for PE in patients with elevated troponin I and hypokinetic dilated right ventricle on echocardiogram. Also if a patient's symptoms do not improve after an intervention or treatment, a second diagnosis must be addressed prior to discharge.
